# Training in childhood obesity management in the United States: a survey of pediatric, internal medicine-pediatrics and family medicine residency program directors

**DOI:** 10.1186/1472-6920-10-18

**Published:** 2010-02-17

**Authors:** Margaret S Wolff, Erinn T Rhodes, David S Ludwig

**Affiliations:** 1 Division of Emergency Medicine, The Children's Hospital of Philadelphia, Philadelphia, PA, USA; 2 Division of Endocrinology, Children's Hospital Boston, Boston, MA, USA; 3 Department of Pediatrics, Harvard Medical School, Boston, MA, USA

## Abstract

**Background:**

Information about the availability and effectiveness of childhood obesity training during residency is limited.

**Methods:**

We surveyed residency program directors from pediatric, internal medicine-pediatrics (IM-Peds), and family medicine residency programs between September 2007 and January 2008 about childhood obesity training offered in their programs.

**Results:**

The response rate was 42.2% (299/709) and ranged by specialty from 40.1% to 45.4%. Overall, 52.5% of respondents felt that childhood obesity training in residency was extremely important, and the majority of programs offered training in aspects of childhood obesity management including prevention (N = 240, 80.3%), diagnosis (N = 282, 94.3%), diagnosis of complications (N = 249, 83.3%), and treatment (N = 242, 80.9%). However, only 18.1% (N = 54) of programs had a formal childhood obesity curriculum with variability across specialties. Specifically, 35.5% of IM-Peds programs had a formal curriculum compared to only 22.6% of pediatric and 13.9% of family medicine programs (p < 0.01). Didactic instruction was the most commonly used training method but was rated as only somewhat effective by 67.9% of respondents using this method. The most frequently cited significant barrier to implementing childhood obesity training was competing curricular demands (58.5%).

**Conclusions:**

While most residents receive training in aspects of childhood obesity management, deficits may exist in training quality with a minority of programs offering a formal childhood obesity curriculum. Given the high prevalence of childhood obesity, a greater emphasis should be placed on development and use of effective training strategies suitable for all specialties training physicians to care for children.

## Background

Childhood obesity is an epidemic problem in the United States. In 2003-2006, approximately one-third of children in the U.S. were overweight or obese [1]. These children are at risk of complications including type 2 diabetes, hypertension, dyslipidemia, and fatty liver [2,3] and increased morbidity as adults [4,5]. However, studies of residents managing children and adults suggest they do not receive adequate training in the management of obesity [6-10] and may underdiagnose and undertreat obesity, feel unprepared for obesity counseling, and desire more training [6,9-15]. Jay et al. surveyed 315 residents and faculty in internal medicine, pediatrics and psychiatry regarding perceived knowledge and skills in obesity management, and almost 20% of respondents reported inadequate competency in every item, and almost half reported an inability to counsel patients about common treatment options [10]. The need for additional training of healthcare providers in childhood obesity prevention and treatment has been highlighted in other studies [16-21], and participants in the first colloquium of the Residency Review and Redesign in Pediatrics Project identified nutrition, especially related to obesity, as an area requiring greater emphasis in pediatric residency training [22]. However, information about the availability or effectiveness of obesity training curricula in pediatric, internal medicine-pediatrics (IM-Peds), or family medicine residency programs is limited [7-9,23].

Perrin et al. demonstrated improvement in confidence, ease and frequency of obesity-related counseling with an intervention for pediatric residents and community pediatricians [23], and Gonzalez et al. demonstrated improvement in resident knowledge, skills and comfort in recognition, evaluation, and management of overweight and obese children and their parents with a curriculum for second year pediatric residents [8]. It has been suggested that a formal curriculum may improve training through structured educational goals, improved faculty support, and acknowledgement of the importance of the subject [24]. In addition, a structured approach may reduce the variability of the training experience [24]. We sought to evaluate the availability of formal childhood obesity curricula in U.S. residency programs training physicians to care for children.

We conducted a survey of all U.S. pediatric, IM-Peds, and family medicine residency program directors to characterize resident training related to childhood obesity. We also sought to assess teaching methods used and their perceived effectiveness, to identify barriers to implementation, and to assess attitudes toward the importance of training residents regarding childhood obesity. We further sought to evaluate whether residency program characteristics were associated with presence of a formal childhood obesity training curriculum. We hypothesized that a minority of pediatric, IM-Peds, and family medicine residency programs in the U.S. would have a formal childhood obesity curriculum but that family medicine programs would be more likely than pediatric programs to have a formal curriculum because of the specialty's emphasis on primary care.

## Methods

### Study Population

The survey was sent to all residency program directors from pediatric, IM-Peds, and family medicine programs in the U.S. identified using the American Academy of Family Physicians public Directory of Family Medicine Residency Programs, the Association of Pediatric Program Directors public database, and the American Medical Association public Fellowship and Residency Electronic Interactive Database (FREIDA) database [25-27]. Only programs with a program director, who could be identified by name, and whose email was specific (i.e., not a generic residency program email) or whose mailing address could be identified, were included. Program directors directing more than one program received only one survey and were asked to complete the survey with respect to the larger program. The study was reviewed and approved by the Committee on Clinical Investigation at Children's Hospital Boston.

### Survey Instrument and Administration

A 24-item survey instrument was developed to address the study aims. Respondents were asked whether their residency program has a formal childhood obesity curriculum, defined as a comprehensive or systematic program that has formal educational goals and either a written curriculum or identified methods for resident education in childhood obesity. Respondents also answered questions regarding resident training in childhood obesity prevention, diagnosis, treatment and diagnosis of complications. Respondents were asked about teaching methods and their perceived effectiveness, barriers to implementation of obesity training, and attitudes toward the importance of childhood obesity training during residency.

Survey development was informed by discussions with childhood obesity experts, primary care physicians managing overweight children, pediatric residency program directors, and a literature review. The survey was pre-tested with assistant residency program directors, a former pediatric residency program director, an internal medicine residency program director, and chief residents at Children's Hospital Boston.

The survey was anonymous and designed for administration via internet or mail. The survey indicated that $1 would be donated toward a campership for a child with type 2 diabetes for each completed survey, and the maximum amount would be donated with receipt of 75% or more completed surveys. A copy of the survey instrument is available upon request.

The survey was fielded between September 2007 and January 2008. Altogether, 711 program directors were sent initial surveys representing 194 (27.3%) pediatric, 76 (10.7%) IM-Peds, and 441 (62.0%) family medicine programs. Initially, 566 (80%) were emailed and 145 (20%) were mailed. Nonrespondents were sent up to three follow-up surveys.

### Analysis

Data are presented as mean and standard deviation or proportions. Bivariate analyses were performed using t-tests, ANOVA, χ^2^, or Fisher's exact. Logistic regression was used to evaluate the relationship between specialty and presence of a formal childhood obesity curriculum. The model was adjusted for program characteristics associated with specialty and presence of a formal childhood obesity curriculum in bivariate analyses at p < 0.2. Respondents who were "unsure" about the presence of a formal curriculum were excluded from analyses with that outcome. For questions regarding methods used for training, a missing response was considered "not used." For barriers to implementation of childhood obesity training, respondents rated items on a scale of 1 to 10 with 1 labeled "not at all a barrier" and 10 labeled "major barrier." We defined a "significant barrier" as a response of 8-10. Statistical significance was considered p < 0.05. Analysis was performed using SAS (Version 9.1, Cary, NC).

## Results

There were 711 surveys fielded. Two program directors had invalid email and mailing addresses, and 299 consented and returned completed surveys. Therefore, the overall response rate was 42.2% (299/709) and ranged by specialty from 40.1% to 45.4%. There were 88 (29.4%) pediatric, 34 (11.4%) IM-Peds, and 176 (58.9%) family medicine programs represented. One respondent (0.3%) did not indicate a specialty. Additional characteristics are presented in Table 1.

**Table 1:** Characteristics of Respondents (N = 299)

	Overall	**Individual Specialties (N = 298)**^**a**^		
		
		Pediatrics	Internal Medicine- Pediatrics	Family Medicine
	N = 299	N = 88	N = 34	N = 176
**Number of Program Residents **^**b**^	28.7 (21.3)	45.7 (31.2)	18.4 (9.5)	22.1 (9.0)
**Community Setting **^**c**^				
Urban	170 (56.9)	63 (71.6)	24 (70.6)	83 (47.2)
Suburban	102 (34.1)	20 (22.7)	6 (17.6)	76 (43.2)
Rural	24 (8.0)	5 (5.7)	4 (11.8)	15 (8.5)
Missing	3 (1.0)	0 (0.0)	0 (0.0)	2 (1.1)
**Setting of Resident Training **^**b**^				
Free-standing children's hospital	80 (26.8)	35 (39.8)	18 (52.9)	27 (15.3)
Department within a hospital	83 (27.8)	35 (39.8)	15 (44.1)	33 (18.8)
Community hospital	109 (36.5)	11 (12.5)	1 (2.9)	97 (55.1)
Military hospital	9 (3.0)	3 (3.4)	0 (0.0)	6 (3.4)
Missing	18 (6.0)	4 (4.5)	0 (0.0)	13 (7.4)
**% of Residents Entering Primary Care **^**b**^				
0-20	9 (3.0)	6 (6.8)	1 (2.9)	2 (1.1)
21-40	16 (5.4)	11 (12.5)	5 (14.7)	0 (0.0)
41-60	61 (20.4)	43 (48.9)	17 (50.0)	1 (0.6)
61-80	39 (13.0)	24 (27.3)	8 (23.5)	7 (4.0)
81-100	174 (58.2)	4 (4.5)	3 (8.8)	166 (94.3)
**US Geographic Region **^**d**^				
Northeast	76 (25.4)	24 (27.3)	9 (26.5)	43 (24.4)
Midwest	89 (29.8)	23 (26.1)	11(32.4)	55 (31.3)
South	81 (27.1)	29 (33.0)	10 (29.4)	42 (23.9)
West	47 (15.7)	10 (11.4)	4 (11.8)	33 (18.8)
Missing	6 (2.0)	2 (2.3)	0 (0.0)	3 (1.7)

Data presented as mean (SD) or N (%).

^a ^One respondent (0.3%) did not indicate a specialty.

Difference across specialties (excluding missing) ^b ^p < 0.0001 ^c ^p < 0.001

^d ^Geographic regions defined by U.S. Census and organized by state of the residency program

### Childhood Obesity Training

Overall, only 18.1% (N = 54) of respondents indicated that their program currently had a formal childhood obesity curriculum. Of these, 79.6% were started in the prior three years. However, most respondents reported resident training in prevention (N = 240, 80.3%), diagnosis (N = 282, 94.3%), diagnosis of complications (N = 249, 83.3%), and treatment (N = 242, 80.9%) of childhood obesity. Overall, one-third (32.8%) reported that residents receive ≤5 hours of childhood obesity training during residency, and 19.4% reported that residents receive >15 hours.

Differences were noted across specialties (Table 2). Whereas 35.5% of IM-Peds programs had a formal childhood obesity curriculum, only 22.6% of pediatric and 13.9% of family medicine programs had a formal curriculum (χ^2^, p < 0.01). IM-Peds programs were more than three times as likely to have a formal childhood obesity curriculum as family medicine programs (OR 3.42, 95% CI: 1.46, 8.01). However, the significance of this relationship was attenuated by adjustment for the proportion of residents entering primary care (OR 4.07, 95% CI: 0.95, 17.42). Pediatric programs were not significantly different from other specialties. In bivariate analyses, U.S. geographic region, community setting, location for majority of resident clinical pediatric training, proportion of residents entering primary care, and the size of the program were not significantly associated with the presence of a formal childhood obesity curriculum.

**Table 2:** Characteristics of Childhood Obesity Training for Residents

	**Pediatrics **^a^		Internal Medicine- **Pediatrics**^a^		**Family Medicine **^a^	
	
Training Characteristic	Sample N	N (%)	Sample N	N (%)	Sample N	N (%)
Formal Childhood Obesity Curriculum ^b ^	84	19 (22.6)	31	11 (35.5)	173	24 (13.9)
Training in Childhood Obesity Prevention	83	70 (84.3)	32	29 (90.6)	170	140 (82.4)
Training in Childhood Obesity Diagnosis	88	84 (95.5)	34	34 (100.0)	174	163 (93.7)
Training in Diagnosis of Complications of Childhood Obesity ^b ^	88	82 (93.2)	32	31 (96.9)	170	135 (79.4)
Training in Childhood Obesity Treatment ^c^	84	74 (88.1)	32	31 (96.9)	170	136 (80.0)

^a ^Sample size varies due to missing data; Difference across specialties, χ^2 ^or Fisher's Exact

^b^p < 0.01 ^c^p < 0.05

For training in individual aspects of obesity management, a significantly lower proportion of family medicine respondents reported training residents in treatment and diagnosis of complications of childhood obesity (Table 2). In addition, 47.1% of IM-Peds programs reported that residents receive >15 hours of childhood obesity training during residency, whereas only 27.3% of pediatric and 9.7% of family medicine programs reported this level of training (χ^2^, p < 0.0001).

### Methods of Training

Among programs offering training in prevention, diagnosis, diagnosis of obesity complications, and/or treatment of childhood obesity, didactic instruction was the most commonly used training method (92.3-97.9%) followed by teaching on inpatient wards (69.5-77.5%). Despite the common use of didactic instruction, 67.9% of respondents using this method rated it as only "somewhat effective," and only 18.9% rated it as "very" or "extremely effective." Compared to other methods, when used, participating in a specialty clinic that focuses on obesity was reported to be "very" or "extremely effective" by the greatest proportion of respondents (59.4%). Additional training methods included precepted patient care in a primary care clinic with focus on obesity, structured individual study with selected reading or educational CD, providing resource lists of texts, providing online materials, elective offerings, and other methods such as community offerings, school-based programs, computer-based education programs, working with a nutritionist, doing obesity-related research, using electronic medical records with prompts for body mass index calculation, attending national obesity conferences, and participation in subspecialty clinics.

### Attitudes and Barriers to Training

Respondents rated the importance of including childhood obesity training in a curriculum for successfully training residents to care for children on a scale from "not at all important" to "extremely important." Overall, 52.5% felt that childhood obesity training was extremely important. However, whereas, 70.6% of IM-Peds respondents rated childhood obesity training as extremely important, only 62.1% of pediatric and 45.9% of family medicine respondents answered similarly (Fisher's exact, p = 0.03). Respondents were then asked to rate, relative to childhood obesity, the importance of required content areas for pediatric residencies identified by the Accreditation Council for Graduate Medical Education (ACGME), which include both specialties and skills. Childhood obesity training was felt to be equally as important as training in advocacy, developmental pediatrics, injury prevention, and school health by at least 50% of respondents and more important than training in genetics, intensive care, and palliative care by at least 50%. Slightly less than half (44.5%) also felt that childhood obesity training was more important than training in subspecialty care.

Overall, the most frequently cited significant barrier to implementing obesity training was other competing curricular demands (58.5%), followed by lack of insurance reimbursement for childhood obesity interventions (44.8%), and inadequate financial resources for program development (40.1%). While these three issues were the top significant barriers in each specialty, their order of importance varied, and other differences across specialties were noted (Table 3). For family medicine programs, lack of training sites to see obese pediatric patients was a significant barrier in a significantly greater proportion of respondents (χ^2^, p = 0.02) compared to the other specialties combined; whereas, lack of administrative support (χ^2^, p = 0.004) and inadequate financial resources for program development (χ^2^, p = 0.02) were significant barriers in a significantly lower proportion.

**Table 3:** Significant Barriers to Implementation of Obesity Training

	**Pediatrics **^**a**^		Internal Medicine- Pediatrics		**Family Medicine **^**a**^	
	
**Barrier **^**b**^	Sample N	N (%)	Sample N	N (%)	Sample N	N (%)
Other competing curricular demands	86	54 (62.8)	34	15 (44.1)	174	106 (60.9)
Lack of insurance reimbursement for childhood obesity interventions	85	40 (47.1)	34	21 (61.8)	172	73 (42.4)
Inadequate financial resources for program development ^c ^	86	42 (48.8)	34	16 (47.1)	174	61 (35.1)
Availability of faculty with experience in childhood obesity treatment and prevention	86	21 (24.4)	34	8 (23.5)	174	42 (24.1)
Lack of administrative support ^d ^	86	23 (26.7)	34	13 (38.2)	172	27 (15.7)
Unclear evidence-base for childhood obesity treatment interventions	86	18 (20.9)	34	5 (14.7)	172	37 (21.5)
Lack of training sites for seeing obese pediatric patients ^c ^	85	9 (10.6)	34	3 (8.8)	174	36 (20.7)
Unclear evidence-base for childhood obesity prevention interventions	86	15 (17.4)	34	5 (14.7)	172	27 (15.7)
Attitudes of faculty regarding importance of childhood obesity	86	2 (2.3)	34	1 (2.9)	174	8 (4.6)
Availability of appropriate patients	86	0 (0.0)	34	0 (0.0)	174	6 (3.5)
Attitudes of residents regarding importance of childhood obesity	86	0 (0.0)	34	0 (0.0)	174	4 (2.3)

Data are proportion of respondents endorsing barrier as "significant" as defined in the Methods section

^a ^Sample size varies due to missing data

^b ^Ordered based on the priority in the overall sample

Difference of Family Medicine vs. Pediatrics and IM-Peds, χ^2 ^^c ^p < 0.05 ^d ^p < 0.01

### Training Evaluation

About half (54.2%) of programs use resident feedback to evaluate childhood obesity training, and a minority use faculty (12.4%) or patient (2.7%) feedback. However, a significantly greater proportion of programs with a formal obesity training curriculum compared to those without use resident feedback (84.9% vs. 48.7%, χ^2 ^p < 0.0001) and faculty surveys (24.0% vs. 10.2%, χ^2 ^p < 0.01).

## Discussion

Studies demonstrating that residents underdiagnose and undertreat childhood obesity and feel unprepared for obesity counseling [10,13] suggest that deficits exist in the quantity and/or quality of U.S. resident training regarding management of childhood obesity. Our findings demonstrate that in a U.S. sample of pediatric, IM-Peds, and family medicine residency programs, the majority do provide resident training in individual aspects of childhood obesity management, such as treatment or prevention. Taken together, this suggests that the availability of resident training in this area may not be a problem but that current approaches may not be effective. Indeed, the most commonly used teaching method, didactic instruction, was rated as only "somewhat effective" by two-thirds of the respondents using this method. In contrast, it has been suggested [24] that a formal curriculum may improve training through structured educational goals, improved faculty support, and acknowledgement of the importance of the subject in ways that an ad hoc approach may not. Only 18.1% of residency programs in this study reported having a formal childhood obesity curriculum. Programs with a formal curriculum, however, were significantly more likely to include training evaluation through resident feedback and faculty surveys, practices in keeping with the recent ACGME focus on evaluation and attainment of objectives in residency education [28]. Studies in other countries [29,30] also highlight training deficits and limited availability of comprehensive resources for training in obesity management.

Expert committee recommendations on childhood obesity management call for a staged treatment approach that relies on the primary care physician to provide most of the initial care [31]. Children with severe obesity that is unresponsive to primary care management or who have comorbid medical problems are then referred to pediatric obesity specialists for further evaluation and management [31]. In the U.S., pediatric endocrinology training must include clinical experience with childhood obesity and obesity-related endocrine disorders [32]. Hyperinsulinemia and prediabetes are common in overweight U.S. adolescents [33] making this expertise relevant to the management of this high risk group. However, Lee et al. reported that the number of U.S. pediatric endocrinologists is insufficient to manage obese children [34], underscoring the need for primary care physicians to be trained in prevention, diagnosis, and treatment of childhood obesity. Many primary care physicians, however, are uncomfortable addressing pediatric obesity and desire more education [18,19]. For example, Jelalian et al. reported that, among physician members of the American Academy of Pediatrics or the American Academy of Family Physicians practicing in Southern New England, one-quarter reported that they were not at all or only slightly competent in addressing childhood obesity [18]. Story et al. also reported that the most common areas of low proficiency in pediatric obesity management for pediatricians and pediatric nurse practitioners were the use of behavioral management strategies and addressing family conflicts [19]. Participants in the first colloquium of the Residency Review and Redesign in Pediatrics Project sponsored by the American Board of Pediatrics Foundation also felt that nutrition, especially related to obesity, should receive greater emphasis in pediatric residency training [22]. Residency training should therefore include a solid foundation in the skills needed to manage this disease; yet few studies [7,8] have assessed specific training in the evaluation and management of childhood obesity offered to residents.

In a qualitative study involving interviews of 16 pediatric residency program directors, Goff et al. reported that limited training was offered in obesity prevention and management despite recognition of obesity as a significant health issue [7]. Further, Gonzalez et al. reported that, among 79 respondents to a questionnaire sent to 200 ACGME accredited pediatric residency training programs, only 17 (21.5%) represented programs offering a structured teaching curriculum on evaluation, management, and counseling of overweight/obese children [8]. Our results are consistent with Gonzalez et al. but extend the findings to a nationwide sample that also includes assessment of residency programs in IM-Peds and family medicine. Similar to Goff et al., we found that slightly more than half of respondents felt that childhood obesity training was extremely important.

While most of our respondents acknowledged the importance of including childhood obesity in residency training, multiple barriers to implementation were cited. The significant barrier endorsed by the greatest proportion was competing curricular demands, suggesting that a successful curriculum needs to efficiently maximize residents' learning. One pilot curriculum described by Gonzalez et al. demonstrated improvement in resident knowledge, skills and comfort in recognition, evaluation, and management of overweight and obese children and their parents [8]. The program was piloted by six second year pediatric residents and included assigned readings, observation of a pediatric nutritionist in a pediatric obesity clinic, problem-based learning cases, and an observed evaluation and counseling session with a patient of the pediatric obesity clinic [8]. Similarly, Perrin et al. evaluated a program for pediatric residents and community pediatricians and demonstrated improvement in confidence, ease and frequency of obesity-related counseling [23]. The program utilized a training session along with supplemental written assessment and counseling tools, and sample case vignettes in written and video format. Though not designed for residents, Hinchman et al. [35] reported use of a program that included two 60-minute interactive training sessions and demonstrated a significant increase in charting of BMI-for-age percentile and using a nutrition and activity self-history form. Recently, Huang et al. proposed the Health and Obesity: Prevention and Education (HOPE) project [36] as a comprehensive web-based curriculum for childhood obesity education targeted at pediatric medical and dental clinicians in training. Other models for education have included programs such as the Centers for Obesity Research and Education (CORE), which provide education and training about obesity to health care professionals through 12 U.S. centers [37]. Future research on the impact of such interventions will inform the optimal intervention.

In our survey, several programs reported using novel approaches, such as computer-based modules. Computer-based modules have been used effectively as a teaching method in medical education [38,39]. However, more research is needed to understand how to optimally integrate novel methods with traditional didactic and clinical teaching. Jay et al. [10], for example, proposed an obesity curriculum based on the 5As framework (assess, advise, agree, assist, arrange) [40]. As multidisciplinary approaches appear to be most successful for pediatric weight management [41], educational curricula will ideally need to incorporate instruction from dietitians, exercise physiologists and mental health providers in order to be comprehensive.

Our survey highlighted that the issues and priorities relevant to resident education may differ across specialties. Such differences were noted in the attitudes, barriers, and teaching methods related to childhood obesity training. Among the specialties, IM-Peds had the greatest proportion of programs with a formal childhood obesity curriculum and, in unadjusted analyses, was significantly more likely than family medicine to have a formal curriculum. Counter to our hypothesis, family medicine programs were least likely to have a formal childhood obesity curriculum despite the fact that, as we anticipated, for 94.3% of these programs, 81-100% of residents enter primary care. Family medicine programs were also least likely to offer training in treatment and diagnosis of complications of childhood obesity. These patterns may be informed by the findings that family medicine had the smallest proportion of respondents endorsing childhood obesity training as extremely important and the highest proportion endorsing lack of training sites for seeing obese pediatric patients as a significant barrier. The opposite was true for IM-Peds programs, which also had a smaller proportion that viewed other competing curricular demands as a significant barrier. Further exploration of attitudes and barriers of professionals in these programs may further inform these findings.

Several limitations merit comment. First, information about nonrespondents was limited to specialty and geographic location, and our response rate was slightly less than half of the sample. As noted previously, response rates among the specialties were similar, and there was no significant difference in the distribution of the three specialties among respondents vs. nonrespondents (data not shown). While the response rate for programs in the Northeast and Midwest were approximately 50%, only approximately one-third of programs in the South and West responded. A response bias may exist if those returning the survey were more likely to be from programs interested in childhood obesity management. If present, such a bias might overestimate the prevalence of residency training related to childhood obesity. Given that only 18.1% of programs reported a formal curriculum, availability of such curricula may be even more limited than suggested here if such a bias were present. With representation from all U.S. geographic regions (Table 1), we found no association between region and availability of a formal curriculum. Nevertheless, future studies to confirm our findings should be considered. Second, in several cases, the survey was completed by a proxy chosen by the residency program director, and we do not have data on the extent of this practice. Although we cannot be certain that program directors' responses would have been the same, the known cases appear to have been chosen because of familiarity with the program's training in obesity. However, as a result of this issue, we did not conduct analyses regarding relationships between respondent characteristics and attitudes toward obesity training. Evaluating and comparing the perspectives of different educators involved in childhood obesity training may further inform the development of training curricula.

## Conclusions

In summary, in a U.S. sample of pediatric, IM-Peds, and family medicine residency programs, we have extended what is known about the availability, methods and perceived effectiveness of resident training programs in childhood obesity. Our findings suggest that, while most residents receive training in some aspects of childhood obesity management, deficits may exist in training quality. Given the high prevalence of childhood obesity in the U.S., greater emphasis should be placed on development of effective training strategies suitable for the multiple specialties that train physicians to care for children.

## Abbreviations

IM-Peds: Internal medicine-pediatrics; FREIDA: Fellowship and Residency Electronic Interactive Database; ACGME: Accreditation Council for Graduate Medical Education

## Competing interests

ER was formerly Chief Medical Officer for Pediatric Weight Management Centers, LLC's Great Moves! Program, which was privately owned and operated in collaboration with the physicians of Children's Hospital Boston. ER neither had nor has any equity or other economic interest in the business. ER also received salary support from an unrestricted, philanthropic grant from the New Balance Foundation to DL at Children's Hospital Boston. MW and DL have no competing interests to disclose.

## Authors' contributions

MW conceived of the study and participated in its design and coordination, the acquisition of data, analysis and interpretation of data, and drafting of the manuscript. ER participated in the study design and coordination, the acquisition of data, analysis and interpretation of data, and drafting of the manuscript. DL participated in the development of the study design, analysis and interpretation of data, and critical revision of the manuscript. All authors have read and approved the final manuscript.

## Pre-publication history

The pre-publication history for this paper can be accessed here:

http://www.biomedcentral.com/1472-6920/10/18/prepub
